# Early Detection of a Carbapenemase-producing organism Outbreak Using Whole Genomic Sequencing

**DOI:** 10.1017/ash.2025.235

**Published:** 2025-09-24

**Authors:** Kim Wright, Mindy Allen, Tyler Parsons

**Affiliations:** 1 Cincinnat Health Department; 2 Cincinnati Health Department

## Abstract

**Background:** In June of 2024 the Cincinnati Health Department Communicable Disease Prevention and Control Unit investigated an outbreak of Carbapenemase-resistant Pseudomonas aeruginosa (CRPA) Verona Integron-encoded Metallo-beta-lactamase (VIM) infections at a local hospital after whole genomic sequencing (WGS) performed by the Centers for Disease Control (CDC) determined two patients had closely related infections. At the time CDC was using WGS to link CRPA infections to the multi-state outbreak associated with artificial tears. CRPA is classified as a Carbapenemase-producing organism (CPO) by the Ohio Department of Health (ODH) and is a Class B reportable disease. According to the ODH Infectious Disease Control Manual, the CPO reportable condition targets organisms that have acquired mobile genetic elements, or plasmids, carrying carbapenemase-producing genes that can be transmitted to other bacteria. CDC’s WGS technology enables faster detection of healthcare-associated infection outbreaks by determining how closely the organisms are related genetically, which facilitates a faster public health response. **Method:** A line list was used to collect data extracted from the patients’ medical records. The hospital was requested to forward all CRPA isolates to the state laboratory for further analysis including WGS at CDC. The facility was provided with cleaning and disinfection guidance and transmission based precaution guidance specific to CRPA. The hospital was also advised to screen roommates of the patients and those who had units in common with the patients. **Result:** Four patients, having a number of other health conditions, with ages ranging from 30-38 (median 35.5) who were hospitalized at the same facility between March and September of 2024 were determined to have closely related CRPA VIM infections through WGS. Their infections were not closely related to the artificial tears-associated outbreak. There were procedural, staff, and potential equipment overlaps found between cases 1 and 2 including Ultrasound-guided ART line and midline performed on consecutive days, and Echo/TTE performed on the same day by the same provider. Cases 3 and 4 had hospital units in common with cases 1 and 2. 50% of cases had gaps in transmission-based precautions. 50% of patients died. One patient was homeless. The hospital did not perform the recommended screenings. **Conclusion:** The detection of two closely related CRPA VIM cases in a hospital through WGS allowed public health responders to quickly identify an outbreak and work closely with the facility in order to implement organism specific infection control measures that helped contain the spread of an insidious healthcare-associated infection to a total of four cases over six months. The outbreak was determined to be over when no new infections were detected for a period of four weeks.

